# Full Body Surface Coverage with Water-Equivalent Bolus as Novel Technique for Total Body Irradiation before Hematopoietic Stem Cell Transplantation in Pediatric Acute Lymphoid Leukemia

**DOI:** 10.3390/children9111740

**Published:** 2022-11-12

**Authors:** Andrea Furka, Zsofia Nagy, Imre Szabó, Gábor Fekete, Ágnes Kelemen, Gábor Bolobás, Gábriel Sebők, Tünde Molnár, János Árvai, Ilona Tornyi, László Kostyál, János Révész, Peter Hauser

**Affiliations:** 1 Department of Oncology, Faculty of Medicine, University of Debrecen, 4032 Debrecen, Hungary; 2 Department of Clinical Radiology, Faculty of Health Care, Institute of Practical Methodology and Diagnostics, University of Miskolc, 3526 Miskolc, Hungary; 3 Centre of Clinical Oncology and Radiotherapy, Borsod-Abaúj-Zemplén County Hospital and University Teaching Hospital, 3526 Miskolc, Hungary; 4 Velkey László Child’s Health Center, Borsod-Abaúj-Zemplén County Central Hospital and University Teaching Hospital, 3526 Miskolc, Hungary; 5 Department of Human Genetics, Faculty of Medicine, University of Debrecen, 4032 Debrecen, Hungary; 6 Faculty of Health Sciences, University of Miskolc, 3526 Miskolc, Hungary

**Keywords:** acute lymphoid leukemia (ALL), bone marrow transplantation, conditioning regimen, full body bolus, pediatric, total body irradiation, volumetric modulated arc therapy (VMAT)

## Abstract

Background: Total body irradiation (TBI) 2 × 2 Gy for 3 consecutive days followed by chemotherapy for conditioning pediatric patients with acute lymphoid leukemia (ALL) before bone marrow transplantation is superior to chemo-conditioning alone. The globally used anterior-posterior/posterior-anterior (AP/PA) technique is the most referable method, but volumetric modulated arc therapy (VMAT) with modern linear accelerators is more precise in terms of ensuring better dose distribution, especially for skin, and higher protection of organs at risk, resulting in less side effects. Method: For TBI, a modern VMAT technique was used. Whole-body immobilization in the supine position was performed using a vacuum mattress with a full body coverage, with a water-equivalent bolus of 1 cm thickness. The design goal was to achieve dose inhomogeneity of less than ±10%. Results: From 2020 to 2022, we performed TBI for five pediatric patients with ALL, with full body bolus and VMAT, who later received hematopoietic stem cell transplantation. No acute complications related to TBI were observed during the treatment period with a median follow-up of 1.27 (0.43–2.11) years. Conclusion: Using full body water-equivalent bolus with VMAT for TBI provides a safe method for children with a better organ sparing in the short term follow-up.

## 1. Introduction

Acute lymphoblastic leukemia (ALL) is the most common pediatric malignancy with more than 90% long term survival in well-developed countries [1]. There are several different trials to treat pediatric ALL based on the different stratifications of patients. Allogeneic hematologic stem cell transplantation (HSCT) with a matched family or unrelated donor is the part of primary treatment in certain groups of patients (hypodiploidy (<44 chromosome), high percentage of residual blast count on day 33 in bone marrow by flow cytometry, poor prednisolone response with T-ALL or with pro B-ALL phenotype, with t(4;11) translocation or with high initial white blood cell count (>100,000 G/l), and high risk patients with poor response on day 15) or in the case of recurrence in selected conditions (T-cell relapse with bone marrow involvement, very early (less than 6 months) relapse, early (less than 18 months) isolated bone marrow relapse, relapse after stem cell transplantation and high percentage of residual blast count on day 28 in bone marrow by flow cytometry or certain genetic subgroups) [2,3].

Two main approaches exist as an ALL conditioning regimen before SCT: chemo-conditioning or total body irradiation (TBI) followed by chemotherapy. Radiation therapy is able to achieve adequate dose effects in areas with bad blood supply, the central nervous system, and in testes, where chemotherapy may be less effective [4]. Ionizing radiation causes cell death through several mechanisms, the most common of which is single and double strand DNA breaks. TBI has been a widely used method in the management of numerus hematological malignancies since the 1970s. It is primarily used as a part of the conditioning regimen before hematopoietic stem cell transplantation [5]. Although it is a good and reliable treatment, it has limitations due to high toxicity. For decades, myeloablative TBI was given as a single fraction of 10 Gy combined with cyclophosphamide [6].

Recently, a randomized, controlled, open-label, international, multicenter, phase 3, non-inferiority study, ALL SCTped 2012 FORUM (For Omitting Radiation Under Majority age) study has proved the superiority of the hyperfractioned TBI (2 × 2 Gy per day on -6, -5, -4 days) followed by etoposide 60 mg/kg on day -3, as a conditioning regimen over chemo-conditioning only (fludarabine, thiotepa, and either busulfan or treosulfan) [7].

The setup, techniques, and practices of TBI are heterogeneous in different institutions, although, in most centers, a two-dimensional conventional technique, such as opposing beams, is still the most frequently used. However, today cobalt machines have largely been replaced by linear accelerators at most centers [4]. It is also crucial to protect the selected organs at risk (OARs), such as the lungs and kidneys [8]. The American Association of Physicists in Medicine guidelines in Report No.17 recommends that the entire body should receive a homogenous radiation dose within 10%. This homogeneity can be achieved by using tissue compensators, like boluses, plastic slabs, or other materials [4].

Skin is the largest organ in the body, and it is known for its complex network of cells that perform cellular communication, angiogenesis, and foremost plays an enormous part in the functioning of the immune system. The skin associated immune system (SALT) is located both in the dermis and epidermis of the skin, and it consists of several important types of immunocompetent cells, such as Langerhans cells, melanocytes, dendritic cells, macrophages, and several T-cell types. These immune cells are important in allergic skin reactions, and some inflammatory skin-diseases, such as psoriasis. It has also been proven that dendritic cells, which migrate to the lymph nodes, play a larger role in the whole immune system [9]. For the above-mentioned reasons, the whole skin surface should be considered as a crucial part of planning target volume (PTV) during TBI, otherwise immune cell escape may occur. A proper dose distribution in the whole body and total surface could guarantee optimal radiotherapeutic effect to destroy malignant cells, therefore, resulting in preferable survival outcomes.

Our aim was to introduce a new technique for proper dose distribution with the use of water-equivalent bolus.

## 2. Materials and Methods

After finding suitable donors, pre-TBI tests were carried out by pediatric-oncologists according to the FORUM protocol [7]. Only children who meet all the criteria receive TBI. For TBI, a Varian Vital Beam linear accelerator (Varian Medical Systems Inc., Palo Alto, CA, USA) was used with the volumetric modulated arc therapy (VMAT) technique, since our center has two identical devices operating side by side in twin bunkers.

**Figure 1:**
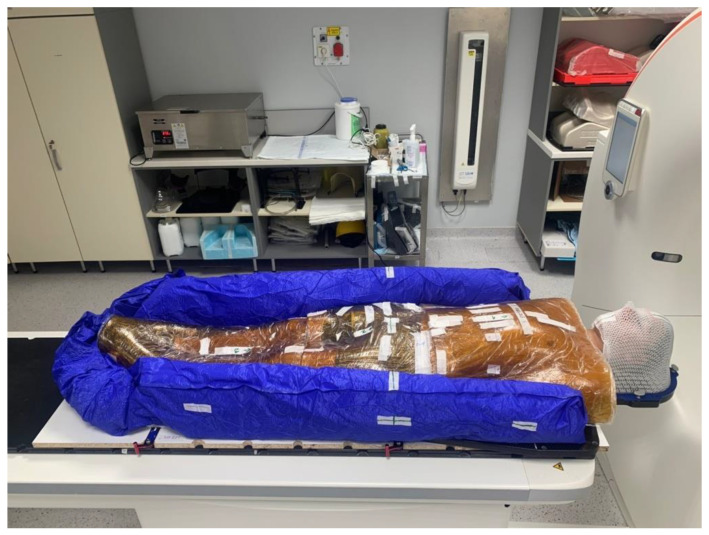
Patients need to be positioned in a reproducible position using a vacuum mattress, head fixation mask, full body bolus, and arrow signed post-its. The reference point is located at mid hip level.

The whole-body immobilization was performed in the supine position using a vacuum mattress with a nearly water-equivalent bolus. The bolus was made of commercially available gelatin (54.35%), glycerin (39.90%), potassium sorbate (0.3%), sodium benzoate (0.3%) and water (11.20%). Its density was 1.08 g/cm^3^. The frontal side of the patient was covered with this bolus (Figure 1). The size of each bolus was 50 cm × 70 cm × 1 cm and 3780 g.

A special vacuum mattress (VacQfix™ Vacuum Cushions, 120 × 230 cm/80 cm, 3-Chamber, Qfix, Avondale, PA, USA) was used for patients comfort and positioning accuracy, which was placed on a 19 mm thick wooden sheet with indexing compatible with the treatment table, so that during the treatments, the patient could be rotated from a head-forward position to a feet-forward position without repositioning the patient. For additional fixation, we also used a thermoplastic head fixation mask (Aquaplast RT™, Variable Perf™, 3.2 mm, Qfix, Avondale, PA, USA), which was also fixed to the treatment table.

To prepare the irradiation plans, we took a whole-body CT scan with a layer thickness of 0.5 cm using a Siemens Somatom Confidence (Siemens Healthcare GmbH, Erlangen, Germany) 20 slices CT. A patient with a maximum height of 182 cm can be imaged in one position without repositioning. This data coincides with the fact that, due to the movement limits of the accelerator table, patients taller than 184 cm cannot be treated even by combining head-forward and feet-forward positions.

**Figure 2:**
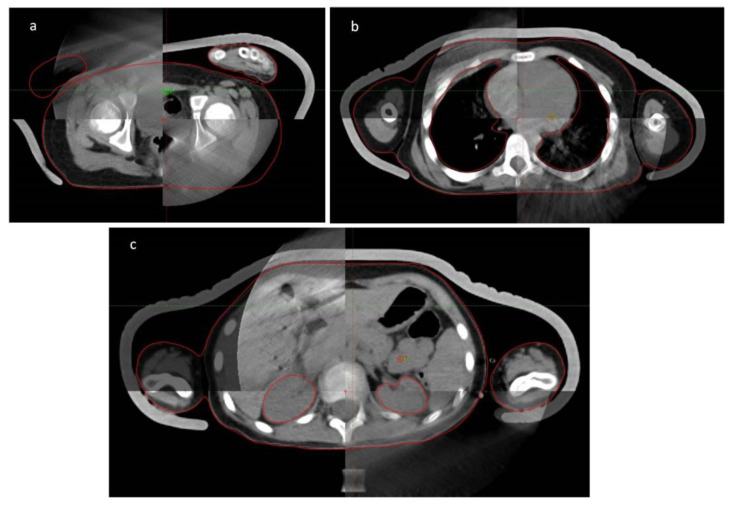
Image guided kilovolt cone-beam CT controls: (**a**) at the isocenter level (**b**) at lung area, (**c**) at kidney area.

The reference point was fixed at approximately half of the patient’s height (Figure 2a).

**Figure 3:**
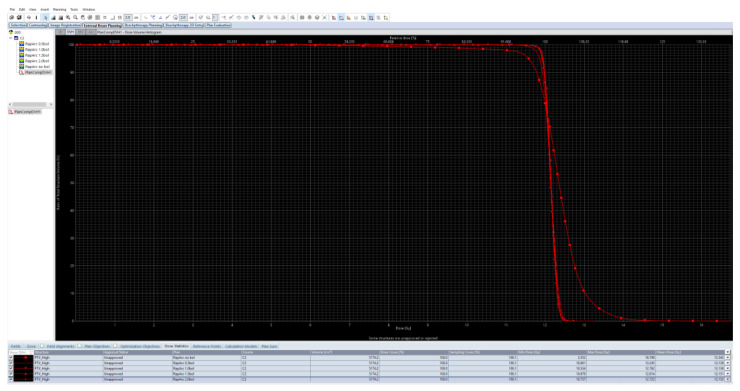
Based on calculations performed on a cylindrical phantom, not only was the coverage insufficient without a bolus, but the maximum dose was also too high. It was also evident that a thickness of 1 cm would be sufficient. Therefore, 1 cm thickness for the water-equivalent bolus was perfect for the phantom. Measured on CT scans, the bolus has a value of 90 ± 20 HU. The thickness and homogeneity of the bolus is extremely constant.

Planning target volume (PTV) was the whole body, including the full outer skin surface. Based on calculations performed on a cylindrical phantom, we determined that the bolus must be at least 1 cm thick (Figure 3).

During contouring, we also included boluses into the body contour. The thickness and material of the laying sheet provided a full-fledged buildup layer, which is also included in the body contour. The PTV is the patient’s entire body including the skin. We did not use an undercut, which is common in the literature, since we also wanted to provide the skin with the prescribed dose.

The prescribed dose was 12 Gy in 2 × 2 Gy fractions per day with a 6 h time gap over 3 days. During irradiation, the megavoltage linear accelerator control unit automatically and continuously optimizes the current dose performance. The usual 6 MV photons were used at a dose rate of up to 120–140 cGy/min. Before the treatments, the plans were checked with portal dosimetry (2%/2 mm), Mobius 3D software (Mobius Medical Systems, Houston, TX, USA), and ionization chamber measurements performed at several points in a cylindrical phantom. As kidneys and lungs cannot receive more than 8 Gy to avoid irreversible life-threatening parenchymal organ dysfunction or failure [10,11], it was ensured that the measurement points fell into the dose ranges corresponding to the lungs and kidneys (Figure 2b,c).

The treatments were planned with the VMAT technique, using Eclipse 15.5 Treatment Planning System (Varian Medical Systems, Palo Alto, CA, USA) and the 15.6.06 algorithm version. The plans had to be divided into 4–8 regions, which corresponded to 9–13 isocenters. In the final optimization, we calculated all sub-plans together. The movements to the individual isocenters were carried out using the delta couch shift option, which enables the most precise positioning. The design goal was to achieve homogeneity of at least ± 10% or better.

At least three cone-beam CT scans were taken during each treatment. The first is the setting of the reference point (Figure 2a), the second is made either in the lung region (Figure 2b) or in the kidney region (Figure 2c). The third is for setting the reference point after turning the patient.

All patients received prophylactic combined antiemetics (ondansetron, dexamethasone). In addition, general anesthesia was used in cases of young children.

## 3. Results

From 2020 to 2022, we performed TBI in five pediatric patients who later received HSCT. The youngest case was 5.45 and the oldest was 16.52 years old. The mean time of each treatment was 50.06 min. Data of our patients and radiotherapy plans are summarized in Table 1 and Table 2.

**Figure 4:**
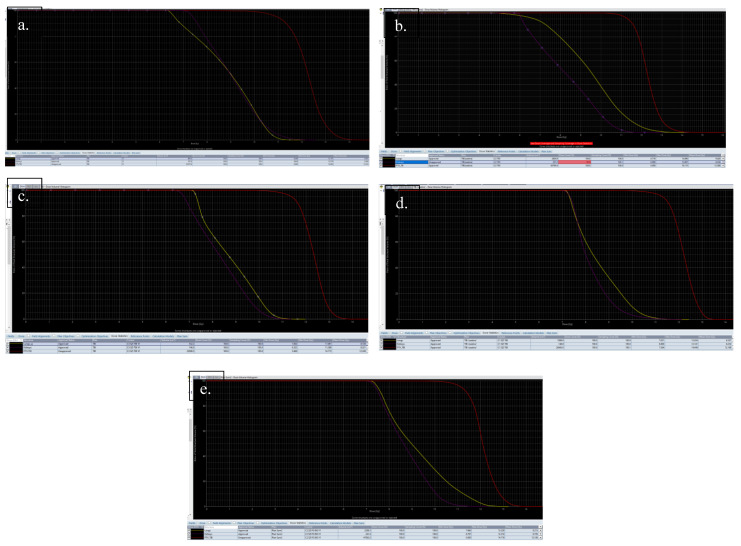
(**a**–**e**): Dose volume histograms of the patients: Patient-1 (**a**), Patient-2 (**b**), Patient-3 (**c**), Patient-4 (**d**), Patient-5 (**e**) (red color: planning target volume (PTV), organ at risks (OAR): yellow color: lungs, lilac color: kidneys).

**Figure 5:**
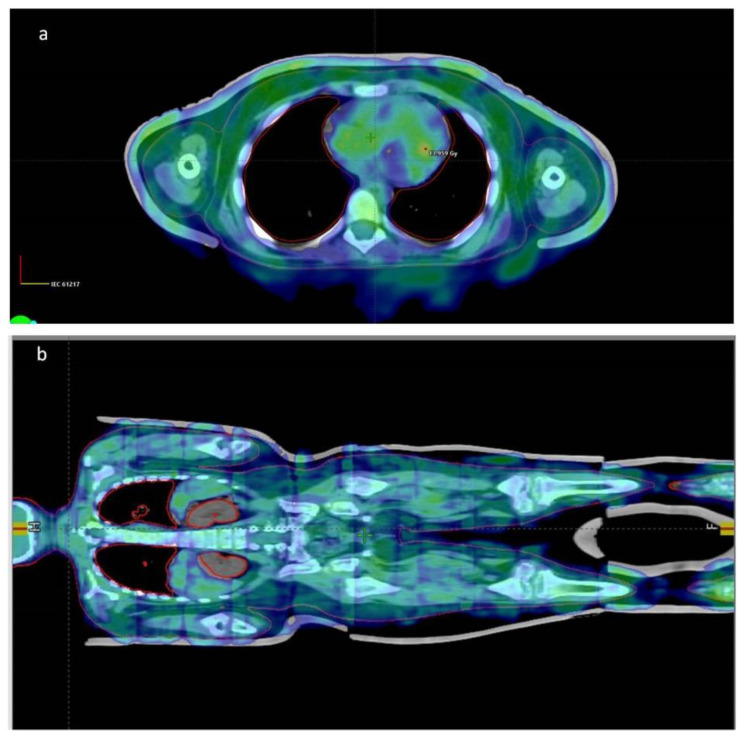
Isoshield dose coverage at 90% dose description on coronal (**a**) and transverse planes (**b**).

The prescribed dose and dose constrain have been feasible for all our patients according to dose volume histograms and isoshield pictures (Figure 4a–e and Figure 5a,b).

No acute complications related to TBI were observed during the treatment period. The most notable abnormality we observed in the subacute phase was radio-mucositis on the tenth day, but this was grade 2–3 and could be successfully treated with local anesthetics and local laser therapy (Heltschl HILARIS TL 50, Heltschl GmbH, Schlüsslberg, Austria). Septic condition also occurred in two cases, but with proper supportive treatment, it was resolved. One patient (Patient 4) developed a secondary graft failure without disease recurrence, which could only be treated with a second hemopoietic stem cell transplantation using the same donor’s cryopreserved peripheral stem cells after a reduced intensity conditioning on Day 135, followed by a donor lymphocyte infusion due to mixed chimerism on Day 81 after the second transplantation. Other subacute adverse events are listed in Table 1. All complications are well-known and are associated to the stem cell transplant procedure, and can be alleviated according to wide-spread supportive care protocols. 

To date, none of our patients have developed any long-term chronic complications, none have developed any permanent, unresolvable damage associated with the treatment, they have all survived the critical 100 days, and they are all well and relapse free.

## 4. Discussion

HSCT using healthy hematopoietic stem cells from a matched related or unrelated donor preceded by a conditioning regimen is a commonly used technique for patients with dysfunctional or depleted bone marrow. It first destroys tumor cells, and then generates functional cells that will replace the dysfunctional ones, depending on the disease being treated. HSCT can be used in non-malignant diseases, such as thalassemia and aplastic anemia, and in malignant diseases, such as AML, ALL, and Hodgkin and non-Hodgkin lymphomas. The first successful bone marrow transplant was performed in monozygotic twins in New York in 1957 (syngeneic transplant) in a patient with acute leukemia by Thomas [12]. According to the FORUM trial in children with ALL over 4 years with indication for HSCT, TBI plus etoposide conditioning is recommended [5,7].

For a long time, skin has been considered a static border between the outside world and the human body, mainly providing protection against dangerous external threats, and allowing sensations of the surrounding world [13]. The so-called “bricks in the wall” theory went through a robust change in the 20th century when many immune reactions were found to be taking place or initiated in the skin [14]. For many years the skin became the target of the majority of laboratory and clinical immune studies, and some authors started using the phrase “skin immune system” [9]. Besides local immune reactions participating in allergic skin reactions and in pathogenesis of some inflammatory skin diseases, such as psoriasis, rosacea, lichen etc., it was found that dendritic cells migrating from the skin to the peripheral lymph nodes and back play a crucial role in orchestrating whole-body immunity [15,16]. In spite of the large surface area, including many permanent and temporary immune cells, the active immunity total body irradiation strategies do not count the proper radiation dose buildup for the skin.

Rassiah et al. conducted a survey among the Children Oncology Group (COG) Centers and found that radiation oncologists perform different TBIs. In 56% of centers, the anterior-posterior/posterior-anterior (AP/PA) technique was the most common technique reported, half of the institutions used the lateral technique, and only 16% used sophisticated techniques, such as volumetric modulated arc therapy or tomotherapy. Almost all responding physicians agreed with the need to refine current TBI techniques, and 79% supported the investigation of new TBI techniques to further lower the lung dose. Unfortunately, there was no concrete protocol in the practice patterns, methods for dose measurement, and reporting of TBI doses among COG institutions. The COG radiation oncology discipline is currently undertaking standardizing procedures regarding the practice and dose reporting of pediatric TBI [4].

We believe that our highly conformal and sophisticated irradiation can only be performed in centers where twin bunkers with identical linear accelerators are available to ensure delivery of the prescribed dose within an optimal time frame and in safe conditions.

The fixation method we have developed is highly reproducible.

During the short term follow-up, VMAT-TBI provides a safe alternative to conventional AP/PA and lateral TBI. The dose modulation capability of VMAT-TBI may lead to new treatment opportunities, such as simultaneous or consecutive boost to the central nervous system, and to better OAR protection, better malignant cell eradication and immune suppression, and lower toxicities [17,18].

Image guided irradiation using an optimized technique ensures safe treatment. Organ shift and intra-fraction motion could be detected with cone-beam CT scans performed before treatment, between isocenter shifts, and after radiation. Therefore, favorable acute and subacute toxicity profiles and excellent disease controls can be achieved [19].

## 5. Conclusions

Comprehensive reporting of innovative conformal TBI techniques, such as the use of full body surface coverage with water-equivalent bolus TBI in conjunction with the VMAT technique, may be a standard procedure in the near future taking, into account the function of the skin immunity. We also know that planning systems cannot accurately calculate the dose in the buildup range. For this reason, it is important to use a bolus to help calculate dosage ranges: the dosage that falls into the bolus can be calculated as that which would otherwise build up in the skin surface and the tissues immediately below it, and thus more accurate dosage ranges can be calculated. The above prescribed technique for TBI may have a potential benefit and better response rate in the preconditioning of pediatric bone marrow transplant cases with longer overall survival rates. Based on the fact that all of our patients survived the critical first 100 days (stem cell transplantation was successful) it suggests that our newly implement full body bolus TBI technique method is safe during the short term follow-up, and is effective without significant acute toxicity. Further investigations and longer follow-up periods are required to compare the overall disease-free survival period and overall survival time to that of conventional techniques.

## Figures and Tables

**Table 1:** Data of the treated patients from the view of pediatrician, indications, last previous treatments, infused cell (CD34/kg), donor HLA matching, biometric data of patients, GVDH prophylaxis, time to engraftment from HSCT, adverse events, conditioning regimen, follow-up time. (AE: adverse event, ALL: acute lymphoblastic leukemia, ALL-IC REL: The Acute Lymphoblastic Leukemia Intercontinental Study Group Relapse Guidance, BKV: BK virus, BM: bone marrow, CML: chronic myeloid leukemia, CMV: cytomegalovirus, CNS: central nervous system, CSA: cyclosporin A, GVHD: graft versus host disease, Gr: Grade, HLA: human leukocyte antigen, HR: high risk, HSCT: hematopoietic stem cell transplant, MTX: methotrexate, PBSC: peripheral blood stem cells, pts: patients, TBI: total body irradiation).

Data of the Treated Patients	Patient-1	Patient-2	Patient-3	Patient-4	Patient-5
Indication	pre-B ALL 2nd relapse, ETV6-RUNX1 (t12;21), CNS 1	T-ALL HR, STIL-TAL1 fusion, CNS 3	B-ALL early relapse, chr 21 tetrasomy, RUNX1 signal overexpression, CNS 1	CML with 2 ALL blastic phase, bcr/abl positive, CNS 1	pre-B-cell ALL relapse bcr/abl positive, CNS 1
Last previous treatment	ALL-IC REL 2016-HR + blinatumomab	ALL-IC BFM 2009	ALL-IC REL 2016 + blinatumomab	ALL-IC REL 2016 + dasatinib, blinatumomab	ALL-IC REL 2016 + dasatinib
Number of administered CD34 positive (cells /kg)	9.2 × 10^6^	0.8 × 10^6^	3.64 × 10^6^	8 × 10^6^	5 × 10^6^
Donor type (HLA)	Matched unrelated donor (9/10)	Matched family donor (10/10)	Matched unrelated donor (12/12)	Matched unrelated donor (11/12)	Matched unrelated donor (10/12)
Height of pts (cm)	134	178	108	131	158
Body weight of pts (kg)	36.4	67	24	27.7	53
Age at transplant of pts (yrs)	10.02	16.52	5.45	8.75	17.39
Gender	Female	Male	Male	Male	Male
GVHD prophylaxis	CSA, MTX	CSA, MTX	CSA, MTX	CSA, MTX	CSA, MTX
Time to neutrophil engraftment	Day + 19	Day + 18	Day + 27	Day + 23	Day + 21
Adverse events	Perianal abscess (Gr3), mucositis (Gr3) sepsis (Gr3)	Mucositis (Gr3), sepsis (Gr2)	Mucositis (Gr2), sepsis (Gr2), Adenovirus viraemia	Secondary graft failure *, Adenovirus and CMV viraemia	Seizure (Gr3), mucositis (Gr4), bowel acute GVHD (Gr4), BKV and Adenovirus related (Gr2) haemorrhagic cystitis and viremia
Conditioning regimen	ALL PED SCT Forum (TBI, etoposide)	ALL PED SCT Forum (TBI, etoposide)	ALL PED SCT Forum (TBI, etoposide)	ALL PED SCT Forum (TBI, etoposide)	ALL PED SCT Forum (TBI, etoposide)
Follow-up time (yrs)	2.11	0.69	1.46	1.43	0.43

* Re-transplantation with the same donor’s cryopreserved peripheral blood stem cells was needed on Day 135 after reduced intensity conditioning (fludarabine-cyclophosphamide). One dose of donor lymphocyte infusion was administered on Day 81 after the second transplantation due to mixed chimerism.

**Table 2:** Data of the radiotherapy plans (CBCT: cone-beam computed tomography, Gy: Gray, IMRT: intensity-modulated radiation therapy, MU: monitor unit, VMAT: volumetric modulated arc therapy).

Data of the Radiotherapy Plans					
Time min (min)	43	49	39	39	51
Time max (min)	125	58	49	44	61
Time avg (min)	60.16	53.17	43	40.33	53.66
CBCT numbers	18	18	18	18	18
Kidneys mean dose (Gy)	9.034	8.256	8.321	8.232	8.726
Lungs mean dose (Gy)	8.851	9.42	8.726	8.537	9.215
Iso centers	9	13	9	10	12
Technic	VMAT	VMAT	VMAT	VMAT	VMAT/IMRT
Arcs/Fields	18	24	18	20	14 Field IMRT + 19
Sum MU	2655.4	4987.1	4449	4094.5	6483.8
Energy	6x photon	6x photon	6x photon	6x photon	6x photon

## Data Availability

The data presented in this study are available on request from the corresponding author.

## References

[1] Pui C.H., Yang J.J., Hunger S.P., Pieters R., Schrappe M., Biondi A., Vora A., Baruchel A., Silverman L.B., Schmiegelow K. (2015). Childhood acute lymphoblastic leukemia: Progress through collaboration. J. Clin. Oncol..

[2] Radu L.E., Colita A., Pasca S., Tomuleasa C., Popa C., Serban C., Gheorghe A., Serbanica A., Jercan C., Marcu A. (2020). Day 15 and day 33 minimal residual disease assessment for acute lymphoblastic leukemia patients treated according to the BFM ALL IC 2009 protocol: Single-center experience of 133 cases. Front. Oncol..

[3] McNeer J.L., Devidas M., Dai Y., Carroll A.J., Heerema N.A., Gastier-Foster J.M., Kahwash S.B., Borowitz M.J., Wood B.L., Larsen E. (2019). Hematopoietic stem-cell transplantation does not improve the poor outcome of children with hypodiploid acute lymphoblastic leukemia: A report from Children’s Oncology Group. J. Clin. Oncol..

[4] Rassiah P., Esiashvili N., Olch A.J., Hua C.H., Ulin K., Molineu A., Marcus K., Gopalakrishnan M., Pillai S., Kovalchuk N. (2021). Practice patterns of pediatric total body irradiation techniques: A children’s oncology group survey. Int. J. Radiat. Oncol. Biol. Phys..

[5] Hoeben B.A., Wong J.Y., Fog L.S., Losert C., Filippi A.R., Bentzen S.M., Balduzzi A., Specht L. (2021). Total Body Irradiation in Haematopoietic Stem Cell Transplantation for Paediatric Acute Lymphoblastic Leukaemia: Review of the Literature and Future Directions. Front. Pediatr..

[6] Kelsey C.R., Horwitz M.E., Chino J.P., Craciunescu O., Steffey B., Folz R.J., Chao N.J., Rizzieri D.A., Marks L.B. (2011). Severe pulmonary toxicity after myeloablative conditioning using total body irradiation: An assessment of risk factors. Int. J. Radiat. Oncol. Biol. Phys..

[7] Peters C., Dalle J.H., Locatelli F., Poetschger U., Sedlacek P., Buechner J., Shaw P.J., Staciuk R., Ifversen M., Pichler H. (2021). Total body irradiation or chemotherapy conditioning in childhood ALL: A multinational, randomized, noninferiority phase III study. J. Clin. Oncol..

[8] Yaray K., Damulira E. (2021). Evaluation of volumetric modulated arc therapy (VMAT)—Based total body irradiation (TBI) in pediatric patients. Rep. Pract. Oncol. Radiother..

[9] Matejuk A. (2018). Skin immunity. Arch. Immunol. Ther. Exp..

[10] Vogel J., Hui S., Hua C.H., Dusenbery K., Rassiah P., Kalapurakal J., Constine L., Esiashvili N. (2021). Pulmonary toxicity after total body irradiation–critical review of the literature and recommendations for toxicity reporting. Front. Oncol..

[11] Klaus R., Niyazi M., Lange-Sperandio B. (2021). Radiation-induced kidney toxicity: Molecular and cellular pathogenesis. Radiat. Oncol..

[12] Khaddour K., Hana C.K., Mewawalla P. (2021). Hematopoietic stem cell transplantation. StatPearls.

[13] Dąbrowska A.K., Spano F., Derler S., Adlhart C., Spencer N.D., Rossi R.M. (2018). The relationship between skin function, barrier properties, and body-dependent factors. Skin Res. Technol..

[14] Kabashima K., Honda T., Ginhoux F., Egawa G. (2019). The immunological anatomy of the skin. Nat. Rev. Immunol..

[15] Ronchese F., Hilligan K.L., Mayer J.U. (2020). Dendritic cells and the skin environment. Curr. Opin. Immunol..

[16] Nakamizo S., Dutertre C.A., Khalilnezhad A., Zhang X.M., Lim S., Lum J., Koh G., Foong C., Yong P.J.A., Tan K.J. (2021). Single-cell analysis of human skin identifies CD14+ type 3 dendritic cells co-producing IL1B and IL23A in psoriasis. J. Exp. Med..

[17] Zhang-Velten E.R., Parsons D., Lee P., Chambers E., Abdulrahman R., Desai N.B., Dan T., Wardak Z., Timmerman R., Vusirikala M. (2022). Volumetric modulated arc therapy enabled total body irradiation (VMAT-TBI): Six-year clinical experience and treatment outcomes. Transplant. Cell. Ther..

[18] Loginova A.A., Tovmasian D.A., Lisovskaya A.O., Kobyzeva D.A., Maschan M.A., Chernyaev A.P., Egorov O.B., Nechesnyuk A.V. (2022). Optimized Conformal Total Body Irradiation methods with Helical TomoTherapy and Elekta VMAT: Implementation, Imaging, Planning and Dose Delivery for Pediatric Patients. Front. Oncol..

[19] Marquez C., Hui C., Simiele E., Blomain E., Oh J., Bertaina A., Klein O., Shyr D., Jiang A., Hoppe R.T. (2022). Volumetric modulated arc therapy total body irradiation in pediatric and adolescent/young adult patients undergoing stem cell transplantation: Early outcomes and toxicities. Pediatr. Blood Cancer..

